# Genome sequences and SNP analyses of *Corynespora cassiicola* from cotton and soybean in the southeastern United States reveal limited diversity

**DOI:** 10.1371/journal.pone.0184908

**Published:** 2017-09-14

**Authors:** Sandesh K. Shrestha, Kurt Lamour, Heather Young-Kelly

**Affiliations:** 1 Department of Entomology and Plant Pathology, The University of Tennessee, Knoxville, TN, United States of America; 2 Department of Entomology and Plant Pathology, The University of Tennessee, Jackson, TN, United States of America; University of Nebraska-Lincoln, UNITED STATES

## Abstract

*Corynespora cassiicola* attackes diverse agriculturally important plants, including soybean and cotton, in the US. It is a reemerge pathogen on cotton in southeastern US. Whole genome sequences of four cotton and one soybean isolate from Tennessee were used to develop single nucleotide polymorphism markers for cotton isolates. Cotton isolates had little diversity at the genome level and very little differentiation from the soybean isolate. Analysis of 75 isolates from cotton and soybean, using targeted-sequencing of 22 polymorphic SNP sites, revealed eight multi-locus genotypes and it appears a single clonal lineage predominates across the southeastern region. The cotton and soybean genome sequences were significantly different from the public reference genome derived from a rubber isolate and the utility of these novel resources will be discussed.

## Introduction

*Corynespora cassiicola* (Berk. & M. A. Curtis) C. T. Wei, first described in 1868 as *Helminthosporium cassicola*, is a pathogen of many crops [1, 2]. It is an anamorphic fungus in the order *Dothideomycetes* in the phylum *Ascomycota* [3]. *C*. *cassiicola* is found on or within 530 plant species from 380 genera—including dicot, monocot, fern and cycad hosts and acts as a pathogen, saprophyte or endophyte [2]. As a pathogen, *C*. *cassiicola* infects plant leaves, stem, and roots; and has been isolated from nematodes and a human corneal infection [4, 5]. Pathogenicity varies depending on the host and some isolates can infect multiple hosts while others appear to be host specific. Isolates recovered from cucumber, green pepper and hydrangea can infect scarlet sage leaves, but not vice versa [6]. Isolates recovered from papaya leaf debris caused leaf lesions on tomato, cucumber, and watermelon but are not pathogenic to papaya [7].

*C*. *cassiicola* attacks soybean [8], cotton [9], tomato [10], cucumber [11], eggplant [12], rubber [13], papaya [14], sweet pepper [15], basil [16], bean [17] and ornamental plants [18]. It has been suggested as a potential biocontrol agent to control noxious weeds (e.g. *Lantana camara*) [19] and exotic invasive weeds (e.g. Brazilian pepper tree in tropical and sub-tropical regions in Florida, Hawaii and Australia) [20].

In the southern US, *Corynespora cassiicola* attacks soybean and cotton causing the foliar disease known as target spot. In soybean, it can also attack roots and the hypocotyls of seedlings [8, 21]. Target spot is present in multiple soybean growing areas in the U.S. The disease is more common in humid condition. The initial visible symptom is a small reddish spot which expands into a circular or irregular reddish-brown lesion, 4–5 mm in diameter, with a targeted or zonate-pattern [22, 23]. In South Carolina, yields were reduced 20% to 40% in a soybean variety field trial [22]. In 2006, among the top eight soybean producing countries, Bolivia and Argentina had the highest estimated yield losses at 500 and 45.3 thousand metric tons, respectively [24]. In 2000, Louisiana had an estimated yield loss of around 11,430 metric tons [25]. Similarly, target spot can cause significant damage to cotton leaves resulting in premature defoliation [26]. Target spot is an emerging pathogen of cotton in the Southeastern US and has been reported in Georgia, Alabama, Louisiana, Mississippi, Arkansas, North Carolina and recently in Tennessee [9, 21, 27–29]. In highly susceptible cultivars, premature defoliation, starting from the lower canopy, can reach up to 75% and reduce the yield of seed cotton by 336 kg/ha [9]. *C*. *cassiicola* causes Corynespora Leaf Fall disease of rubber and the levels of a putative effector protein, cassiicolin, differ between aggressive and moderately aggressive isolates [30]. Investigation of the cassiicolin gene for diverse isolates revealed significant variation and may be related to host range [31].

Random amplified polymorphic DNA (RAPD) markers differentiated isolates from diverse locations and hosts although a clonal lineage from rubber was not correlated with host or location [32–36]. Investigations using the ITS region and other genes showed no correlation between geographical location, although in some cases there was a correlation with the host [4, 37].

Our goal was to develop genetic resources for isolates of *C*. *cassiicola* from Tennessee, particularly for cotton, and to investigate genotypic diversity for isolates recovered from cotton and soybean in Tennessee and surrounding states.

## Materials and methods

### Sample recovery and DNA extraction

Permission to collect samples was received from all land owners. Leaves with typical symptoms of target spot were surface sterilized with 10% chlorine for 1 min and a section of tissue at the edge of a lesion was excised and placed onto RA-amended water agar media (rifampicin 25 ppm, ampicillin 100 ppm, 20 g agar and 1 L water). Hyphal-tips were transferred to RA-amended V8 agar media (15 g agar, 3 g calcium carbonate + 160 mL V8 juice + 840 mL water) and maintained at -4 °C.

For genomic DNA extraction for Whole Genome Sequencing (WGS), mycelium was grown 2 weeks at room temperature in 250 ml flasks containing 10 ml RA-V8 liquid broth (above, minus agar). The resulting mycelium was transferred to 2 ml tubes containing 2–3 glass beads, freeze dried, and powdered with a Mixer-Mill (Qiagen). Genomic DNA was extracted using a standard phenol-chloroform protocol. DNA extraction for targeted-sequencing was accomplished in a 96 well plate as described by Lamour and Finley [38].

### Whole genome sequencing

Isolates selected for WGS were confirmed by sequencing the internal transcribed spacer (ITS) using the ITS5 (5’ GGAAGTAAAAGTCGTAACAAGG 3’) and ITS4 (5’ TCCTCCGCTTATTGATATGC 3’) primers as previously described [39]. High-quality genomic DNA was sheared with a Bioruptor Plus device (Diagenode, Inc.). Briefly, genomic DNA was diluted to 10 ng/μl with TE (10 mM Tris, 1mM EDTA, pH 7.5–8.0 buffer) and 100 μl was transferred to 0.5 ml Bioruptor microtubes (Diagenode, Inc.). The samples were incubated on ice for 15 minutes and sheared with the following setting: on/off-30/90 sec for 30 cycles. The fragmented DNA was visualized on a 2% gel and 200–300 bp fragments excised and cleaned using a PureLink Quick Gel Extraction Kit (Thermo Fisher Scientific Inc.). Illumina libraries were prepared using a PCR-free KAPA Hyper Prep Kit followed by qPCR library quantitation using the KAPA Library Quantification Kit (Kapa Biosystems) and sequenced on an Illumina device. Raw sequences were deposited in National Center for Biotechnology Information (NCBI) database as BioProject (PRJNA382361).

### Genome comparison of isolates from cotton and soybean to an isolate from rubber

Raw FASTQ files were quality trimmed with FASTQC and Trimmomatic version 0.33 [40, 41]. Reads were mapped using CLC Genomics Workbench (Qiagen) to the public *C*. *cassiicola* genome sequence which is derived from an isolate recovered from rubber (http://genome.jgi.doe.gov/Corca1/Corca1.home.html). Resulting BAM files were processed using GATK to identify putative SNP positions [42]. Sequences were mapped requiring 90% of the sequence matches at least 90% of the reference genome. Variant calling was done with HaplotypeCaller at default settings for the haploid genome. After recommended hard filtering, SNP genotypes were assigned using custom Perl scripts (https://github.com/sandeshsth) to require a minimum of 10X and maximum of 1000X coverage and an alternate allele frequency of 100%. The impact of putative SNPs was assessed using SnpEFF [43].

### Marker development for differentiating cotton isolates

To identify SNPs useful on cotton (and possibly soybean) in the southeastern region, TS_cotton1 was *de novo* assembled using CLC Genomics Workbench and the resulting contigs were used as a reference for mapping the cotton and soybean isolates. Candidate variants were identified with an alternate allele frequency of 100%. Custom Perl scripts (https://github.com/sandeshsth/) were used to annotate the reference contigs and target regions (100bp on each side of the target SNP) were extracted and used to design general PCR primers using Batchprimer3 [44]. Multiplex amplification of the targets was done by Floodlight Genomics, LLC (Knoxville, TN) to produce sample-specific amplicons using an optimized Hi-Plex approach as part of a no-cost Educational and Research Outreach Program [45]. Pooled barcoded amplicons were sequenced on a HiSeq3000 device and the sample-specific sequences were aligned to the sequences used for primer design with CLC Genomics Workbench and genotypes assigned using GATK (>10X coverage and 100% alternate allele).

## Results

### Isolates

Sixty-five isolates were recovered from 15 cotton cultivars planted at the West Tennessee Research and Education Center in Jackson, TN in 2015. An additional ten isolates from cotton and soybean from Florida, Louisiana, Georgia and Virginia were also included in the study (Table 1). The year of isolation for these isolates was unknown but was prior to 2015.

**Table 1:** Summary data for *C*. *cassiicola* isolates including host, cultivar (if known), number of isolates, genotypes and location.

Host	Cultivars	No. of Isolates	Genotypes*	Location
Cotton	Bayer Stoneville ST5032GLT	9	G1(6), G2(2), G6(1)	TN
Cotton	Bayer Stoneville ST4747GLB2	3	G1(3)	TN
Cotton	Bayer Bx 1532 GLT	6	G1(5), G2(1)	TN
Cotton	Dyna-Gro DG 2570 B2RF	7	G1(4), G2(1), G3(1), G5(1)	TN
Cotton	Phytogen 312	7	G1(5), G2(1), G7(1)	TN
Cotton	Bayer Stoneville ST4946GLB2	4	G1(2), G3(2)	TN
Cotton	Phytogen 333	2	G4(1), G8(1)	TN
Cotton	Bayer Bx 1630GLT	2	G2(1), G4(1)	TN
Cotton	Phytogen 444	5	G1(4), G2(1)	TN
Cotton	Phytogen 222	4	G1(2), G4(1), G5(1)	TN
Cotton	Bayer Bx 1531GLT	6	G1(3), G2(3)	TN
Cotton	Bayer Stoneville ST6182GLT	3	G1(3)	TN
Cotton	Bayer Bx 1633 GLT	2	G1(2)	TN
Cotton	Phytogen 427	3	G1(2), G2(1)	TN
Cotton	Phytogen 499	2	G1(2)	TN
Cotton		1	G1(1)	FL
Cotton		1	G1(1)	GA
Cotton		1	G1(1)	GA
Cotton		1	G1(1)	VA
Cotton		1	G1(1)	LA
Soybean		1	G1(1)	GA
Soybean		1	G1(1)	GA
Soybean		1	G1(1)	GA
Soybean		1	G1(1)	GA
Soybean		1	G1(1)	GA
Total		75		

*The number of isolates with one of the eight described multi-locus genotypes is denoted in parentheses.

### Whole genome sequences

Five isolates of *C*. *cassiicola* from Tennessee were selected for WGS, including four from cotton and one from soybean. At the time of sequencing we did not have access to isolates from surrounding states. Isolates from cotton included an isolate from Jackson, TN recovered in 2013 (used to report the first occurrence of target spot on cotton in Tennessee) and three isolates recovered from cotton in Jackson, TN in 2015 [29]. The isolate from soybean was recovered from Jackson, TN in 2015. Isolates are named TS_cotton1 (2013), TS_cottton2, TS_cottton3, TS_cottton4 and TS_soybean.

An initial comparison of the genome sequences for the cotton isolates indicates they are essentially identical and the TS_cottton1 (2013), TS_cottton2 and TS_soybean isolates were analyzed further to identify SNP sites and determine overall metrics. After quality trimming, TS_cotton1 (2013), TS_cotton2, and TS_soybean had approximately 43, 8 and 6 million paired-end reads, respectively. In total, 80.4% (TS_cotton1), 70.8% (TS_cotton2), and 78.28% (TS_soybean) of the reads mapped to the rubber isolate reference genome. Greater than 95% of the annotated genes in the rubber isolate reference genome are covered. Analysis using GATK identified 807,433 variable sites of which >99% were fixed differences between the cotton and the soybean isolates compared to the isolate from rubber. Comparison of the two cotton isolates revealed 16 putative SNP sites and comparison between cotton and soybean revealed 1627 candidate SNP sites. For the 807K variable sites (between the cotton + soybean isolates and the rubber isolate), 30% are predicted to be missense and 25% silent mutations.

### SNP marker development and application

*De novo* assembly of TS_cotton1 (2013) produced 1846 contigs with a total size of about 42Mbp, similar to the 44.5 Mbp genome available for the rubber isolate. The other three cotton isolates were mapped to the 1846 contigs and 82.7%, 96.5% and 95.3% of the reads from TS_cotton2, TS_cotton3 and TS_cotton4 mapped, respectively. A total of 408 Single Nucleotide Variant (SNV) were discovered and from these, a subset of 40 SNV’s from different contigs were selected for targeted sequencing and assessment in field populations.

A total of 22 SNP markers in 75 isolates of *C*. *cassiicola* were retained for analysis after removing all monomorphic markers and missing data; revealing eight unique multi-locus genotypes (Table 2, Table 3). Genotypes are assigned from G1 to G8. The G1 genotype was the most frequent and dominated the populations recovered from cotton in TN and included all ten isolates from the other states.

**Table 2:** Summary data for single nucleotide polymorphism (SNP) markers.

SNP ID	Contig_position	REF	ALT	Forward 5'-3'	Reverse 5'-3'
S1	C.cassiicola_43_171974	G	A	GCAGCTAACGCCAATCG	TGTGCGAGGCCGTGTA
S2	C.cassiicola_57_41402	T	G	GCTCAATGGTCCACCACA	AAATAAGGCACGCCTCAAGA
S3	C.cassiicola_89_187187	G	A	CCGGCGTCGTCGTTG	CGGCCATGGACCTCAA
S4	C.cassiicola_96_120020	C	T	TGCCAATGCATGTTCTGC	GCTGGGGAGCACAAGG
S5	C.cassiicola_149_87438	C	T	CGCGATATCCACGTCTCA	CCGGCCAATGAGGTGA
S6	C.cassiicola_227_2218	C	A	GGTAGTCTTCCCAATTTATTTCG	TTGATGTCCTCAAAAACTCCAA
S7	C.cassiicola_242_152649	T	C	CTGTGCCGGGCTCATC	GGAGGGGAACGGCGTA
S8	C.cassiicola_251_87513	A	G	TGCATTTTACGTCTTCATGTTTG	CACGGCTCCACACCTCA
S9	C.cassiicola_283_24893	A	G	TCTCGCCAGACCAAAGAAA	TCCCCTTGAAATAGCATGA
S10	C.cassiicola_434_232485	T	C	CGTTCATGAAAGCCAACG	CGACTGGCGGCTGAGT
S11	C.cassiicola_504_40819	A	T	CAAGGCGCTACGTCGTC	TGGGTAGGGATGCCAGTC
S12	C.cassiicola_558_27717	A	G	ACTGCTTCCCTCCACGAG	CAACATTCCCGATACAAAACG
S13	C.cassiicola_571_22394	G	A	TCAACGCGCATGCAAC	TGCATTGACCAGGGTCTG
S14	C.cassiicola_1221_3365	T	C	TGGTGCTATAGCGATAGTTTCCT	AGCTGTTAACGTGTAAGAATGC
S15	C.cassiicola_1_49273	C	T	GAACAACGCTATCTCTTCTTTCG	GAAATTGGCCATCAACTGC
S16	C.cassiicola_56_39229	G	A	CCGATGACAAATGCGGTA	CCCGAAGGCTGGGAAT
S17	C.cassiicola_183_39389	C	A	GCCCCAGTGTCTTTTTCG	GACCAGGACGTCGCATTT
S18	C.cassiicola_391_68335	A	G	CACGCAGGGCTGCAA	CGGCGCGCTTCGAG
S19	C.cassiicola_435_40268	G	A	CGGAGCTCCTCGCTGTT	TGGAGCGCCTCTGATTG
S20	C.cassiicola_519_30398	C	A	AAATGTCAATCAACCAAAACGA	TCTCCTTTTCATTCCAACCAA
S21	C.cassiicola_550_32545	C	T	ACAGGATCGTCGGGAGTG	CAGCGACGATGCTACGC
S22	C.cassiicola_73_17218	T	C	CCTGCGGCGACCAC	GGGTTGCTCTCGGGAAG

**Table 3:** Summary data for the eight unique genotypes of *C*. *cassiicola*. Genotypes are in order, S1 to S22 as presented in Table 2.

Genotype ID	Genotype	Number of isolates
G1	CCCACCAGGGTGACCGGGGCAC	53
G2	CCCACCAGGGTGACCGGGGCGC	11
G3	TCCACCGGGGTAAACGGGGCAC	3
G4	CCCACCAGGATGTCCGGGGCAC	3
G5	CCCACCAGGATGTCCGGGGCAT	2
G6	CCTAACAGGGCGACTGAGACAC	1
G7	CTCCCTAAAGTGACCAGTGTGC	1
G8	CCCACCGGGGTGACCGGGGCAC	1

## Discussion

Our goal was to investigate the genetic diversity of *C*. *cassiicola* recovered from cotton and soybean in Tennessee and to investigate diversity in the southeastern region. Overall, whole genome sequencing revealed almost no differences between four cotton isolates and a limited amount of variation between the cotton isolates and an isolate from soybean. There is some evidence that isolates recovered from cotton and soybean can cause disease on cotton but not on soybean [46]. Pathogenicity test showed that only soybean isolates can cause disease on soybean and isolates from cotton are more aggressive on cotton when compared to isolates from soybean. Further analysis of field isolates using a relatively small set of SNP markers indicates a very low level of genotypic variation, typical for foliar fungal pathogens spread widely as clonal lineages. This could be due to the recent introduction of a highly successful clonal lineage of *C*. *cassiicola* to TN and surrounding states [9, 27, 29]. Development of additional SNP markers, using WGS from a wider array of isolates would be useful and the sequences presented here will be useful in this endeavor.

Although isolates from cotton and soybean were highly similar and had an estimated SNP only every 25,000bp, they were both highly dissimilar to an isolate recovered from rubber with a SNP site every 40bp. We also did a whole genome comparison to an isolate recovered from a contact lens in Malaya (NCBI Bioproject PRJNA236064) and found a similarly high level of dissimilarity with over 1M putative SNPs across 40Mbp of genome sequence (Data not shown).

When compared to the isolate pathogenic to rubber, there were more missense mutations predicted than silent mutations–which supports the notion that these isolates belong to distinct evolutionary lineages that have diverged over an extended period. A previous investigation of *C*. *cassiicola* isolates using four genetic loci placed isolates from rubber and soybean into the same, as well as, different lineages out of six total lineages [4]. Although our work has a limited scope (considering the wide host range of this organism), it suggests that a revision of the genus using whole genome data may be helpful to assign isolates to anamorphic lineages or possibly distinct species.

The limited number of candidate SNP loci identified by WGS suggests a single clone may predominate in the southeastern region. This is not surprising as *C*. *cassiicola* is apparently new to the region and can produce copious airborne spores on foliar lesions. Further work characterizing the pathogen over time will be useful to track the epidemiology and monitor for cryptic sexual recombination and/or the introduction of novel clonal lineages [9, 27, 29, 47].

## References

[1] Wei C. Notes on *Corynespora*. Mycological Papers. 1950;34:1–10.

[2] Smith L. Host range, phylogenetic and pathogenic diversity of Corynespora cassiicola (Berk. & Curt.) Wei. PhD dissertation, University of Florida, Gainesville. 2008.

[3] Schoch C, Crous PW, Groenewald JZ, Boehm E, Burgess TI, De Gruyter J, et al A class-wide phylogenetic assessment of Dothideomycetes. Studies in Mycology. 2009;64:1–15. doi: 10.3114/sim.2009.64.01 20169021 10.3114/sim.2009.64.01 PMC2816964

[4] Dixon L, Schlub R, Pernezny K, Datnoff L. Host specialization and phylogenetic diversity of *Corynespora cassiicola*. Phytopathology. 2009;99(9):1015–27. doi: 10.1094/PHYTO-99-9-1015 19671003 10.1094/PHYTO-99-9-1015

[5] Yamada H, Takahashi N, Hori N, Asano Y, Mochizuki K, Ohkusu K, et al Rare case of fungal keratitis caused by *Corynespora cassiicola*. Journal of Infection and Chemotherapy. 2013;19(6):1167–9. doi: 10.1007/s10156-013-0579-8 23494266 10.1007/s10156-013-0579-8

[6] Furukawa T, Ushiyama K, Kishi K. Corynespora leaf spot of scarlet sage caused by *Corynespora cassiicola*. Journal of General Plant Pathology. 2008;74(2):117–9.

[7] Kingsland GC. Pathogenicity and epidemiology of *Corynespora cassiicola* in the Republic of Seychelles. International Journal of Pest Management. 1986;32(4):283–7.

[8] Seaman W, Shoemaker R, Peterson E. Pathogenicity of *Corynespora cassiicola* on soybean. Canadian Journal of Botany. 1965;43(11):1461–9.

[9] Fulmer A, Walls J, Dutta B, Parkunan V, Brock J, Kemerait R Jr. First report of target spot caused by *Corynespora cassiicola* on cotton in Georgia. Canadian Journal of Plant Pathology. 2014;36:407–11. 10.1094/PDIS-01-12-0035-PDN 30727240

[10] Schlub R, Smith L, Datnoff L, Pernezny K. An overview of target spot of tomato caused by Corynespora cassiicola. II International Symposium on Tomato Diseases 808. 2007:25–8.

[11] Blazquez C. Corynespora leaf spot of cucumber. Proceedings of the Florida State Horticultural Society. 1967;80:177–82.

[12] Shimomoto Y, Kiba A, Hikichi Y. Multiplex polymerase chain reaction discriminates which eggplant isolates of *Corynespora cassiicola* are virulent to sweet pepper. Journal of General Plant Pathology. 2015;81(3):226–31.

[13] AdS Liyanage, C Jayasinghe, N Liyanage A Jayaratne. Corynespora leaf spot disease of rubber (*Hevea brasiliensis*)—a new report. J Rubber Res Inst Sri Lanka. 1986;65:47–50.

[14] Silva W, Wijesundera R, Karunanayake E, Jayasinghe C, Priyanka U. New hosts of *Corynespora cassiicola* in Sri Lanka. Plant Disease. 2000;84(2):202–. 10.1094/PDIS.2000.84.2.202D 30841332

[15] Shimomoto Y, Adachi R, Morita Y, Yano K, Kiba A, Hikichi Y, et al Corynespora blight of sweet pepper (*Capsicum annuum*) caused by *Corynespora cassiicola* (Berk. & Curt.) Wei. Journal of General Plant Pathology. 2008;74(4):335–7.

[16] Garibaldi A, Rapetti S, Rossi J, Gullino M. First report of leaf spot caused by *Corynespora cassiicola* on basil (*Ocimum basilicum*) in Italy. Plant Disease. 2007;91(10):1361–. 10.1094/PDIS-91-10-1361B 30780548

[17] Qi Y-X, Zhang X, Pu J-J, Liu X-M, Lu Y, Zhang H, et al Morphological and molecular analysis of genetic variability within isolates of *Corynespora cassiicola* from different hosts. European Journal of Plant Pathology. 2011;130(1):83–95.

[18] Jayasuriya K, Thennakoon B. First report of *Corynespora cassiicola* on *Codiaeum variegatum* (croton) in Sri Lanka. Ceylon Journal of Science (Biological Sciences). 2007;36(2):138–41.

[19] Pereira JMc Barreto RW, Ellison CA Maffia LA. *Corynespora cassiicola* f. sp. *lantanae*: a potential biocontrol agent from Brazil for *Lantana camara*. Biological Control. 2003;26(1):21–31.

[20] de Macedo DM, Pereira OL, Wheeler GS, Barreto RW. *Corynespora cassiicola* f. sp. *schinii*, a potential biocontrol agent for the weed *Schinus terebinthifolius* in the United States. Plant Disease. 2013;97(4):496–500. 10.1094/PDIS-06-12-0598-RE 30722252

[21] Faske T. Cotton disease alert: Corynespora leaf spot has been detected in Arkansas. Division of Agriculture, University of Arkansas. Available from: http://www.arkansas-crops.com/2013/08/26/cotton-disease-alert-corynespora-leaf-spot-has-been-detected-in-arkansas/. 2015.

[22] Koenning SR, Creswell TC, Dunphy EJ, Sikora EJ, Mueller JD. Increased occurrence of target spot of soybean caused by *Corynespora cassiicola* in the Southeastern United States. Plant Disease. 2006;90(7):974–. doi: 10.1094/PD-90-0974C 10.1094/PD-90-0974C 30781053

[23] Faske T, Kirkpatrick T. Target spot of soybean: What do we know?. Division of Agriculture, University of Arkansas. Available from: http://www.arkansas-crops.com/2016/11/02/target-spot-of-soybean-what-do-we-know/. 2014.

[24] Wrather A, Shannon G, Balardin R, Carregal L, Escobar R, Gupta G, et al Effect of diseases on soybean yield in the top eight producing countries in 2006. Plant Health Progress doi: 10.1094/PHP-2010-0125-01-RS. 2010.

[25] Wrather J, Koenning S, Anderson T. Effect of diseases on soybean yields in the United States and Ontario (1999–2002). Plant Health Progress doi: 10.1094/PHP-2003-0325-01-RV. 2003.

[26] Lakshmanan P, Jeyarajan R, Vidhyasekaran P. A boll rot of cotton caused by *Corynespora Cassiicola* in Tamil Nadu, India. Phytoparasitica. 1990;18(2):171–3. doi: 10.1007/BF02981234

[27] Conner K, Hagan A, Zhang L. First report of *Corynespora cassiicola*-incited Target Spot on cotton in Alabama. Plant Disease. 2013;97(10):1379–. 10.1094/PDIS-02-13-0133-PDN 30722137

[28] Koenning S, Edmisten K. Cotton disease update: leaf spots on cotton, North Carolina State University. Available from: https://cotton.ces.ncsu.edu/2013/08/cotton-disease-update-leaf-spots-on-cot/. 2015.

[29] Butler S, Young-Kelly H, Raper T, Cochran A, Jordan J, Shrestha S, et al First report of Target Spot caused by *Corynespora cassiicola* on cotton in Tennessee. Plant Disease. 2016;100(2):535.

[30] Déon M, Bourré Y, Gimenez S, Berger A, Bieysse D, De Lamotte F, et al Characterization of a cassiicolin-encoding gene from *Corynespora cassiicola*, pathogen of rubber tree (*Hevea brasiliensis*). Plant Science. 2012;185:227–37. doi: 10.1016/j.plantsci.2011.10.017 22325885 10.1016/j.plantsci.2011.10.017

[31] Déon M, Fumanal B, Gimenez S, Bieysse D, Oliveira RR, Shuib SS, et al Diversity of the cassiicolin gene in *Corynespora cassiicola* and relation with the pathogenicity in *Hevea brasiliensis*. Fungal Biology. 2014;118(1):32–47. doi: 10.1016/j.funbio.2013.10.011 24433675 10.1016/j.funbio.2013.10.011

[32] Silva W, Multani D, Deverall B, Lyon B. RFLP and RAPD analyses in the identification and differentiation of isolates of the leaf spot fungus *Corynespora cassiicola*. Australian Journal of Botany. 1995;43(6):609–18.

[33] Silva W, Deverall B, Lyon B. Molecular, physiological and pathological characterization of Corynespora leaf spot fungi from rubber plantations in Sri Lanka. Plant Pathology. 1998;47(3):267–77.

[34] Romruensukharom P, Tragoonrung S, Vanavichit A, Toojinda T. Genetic variability of *Corynespora cassiicola* populations in Thailand. Journal of Rubber Research. 2005;8(1):38–49.

[35] Kurt S. Genetic variation in *Corynespora cassiicola*, the target leaf spot pathogen. Pakistan Journal of Biological Sciences. 2005;8(4):618–21.

[36] Silva WP, Karunanayake EH, Wijesundera RL, Priyanka U. Genetic variation in *Corynespora cassiicola*: a possible relationship between host origin and virulence. Mycological Research. 2003;107(05):567–71. 12884953 10.1017/s0953756203007755

[37] Hieu ND, Nghia NA, Chi VTQ, Dung PT. Genetic diversity and pathogenicity of *Corynespora cassiicola* isolates from rubber trees and other hosts in Vietnam. Journal of Rubber Research. 2014;17(3):187–203.

[38] Lamour K, Finley L. A strategy for recovering high quality genomic DNA from a large number of Phytophthora isolates. Mycologia. 2006;98(3):514–7. 17040080 10.3852/mycologia.98.3.514

[39] White TJ, Bruns T, Lee S, Taylor J. Amplification and direct sequencing of fungal ribosomal RNA genes for phylogenetics. PCR protocols: a guide to methods and applications, Academic Press. 1990;18(1):315–22.

[40] Andrews S. FastQC: a quality control tool for high throughput sequence data. Available from: http://www.bioinformatics.babraham.ac.uk/projects/fastqc/. 2010.

[41] Bolger AM, Lohse M, Usadel B. Trimmomatic: a flexible trimmer for Illumina sequence data. Bioinformatics. 2014;30(15):2114–20. doi: 10.1093/bioinformatics/btu170 24695404 10.1093/bioinformatics/btu170 PMC4103590

[42] McKenna A, Hanna M, Banks E, Sivachenko A, Cibulskis K, Kernytsky A, et al The Genome Analysis Toolkit: a MapReduce framework for analyzing next-generation DNA sequencing data. Genome Research. 2010;20(9):1297–303. doi: 10.1101/gr.107524.110 20644199 10.1101/gr.107524.110 PMC2928508

[43] Cingolani P, Platts A, Wang LL, Coon M, Nguyen T, Wang L, et al A program for annotating and predicting the effects of single nucleotide polymorphisms, SnpEff. Fly. 2012;6(2):80–92. doi: 10.4161/fly.19695 22728672 10.4161/fly.19695 PMC3679285

[44] You FM, Huo N, Gu YQ, Luo M-c, Ma Y, Hane D, et al BatchPrimer3: a high throughput web application for PCR and sequencing primer design. BMC Bioinformatics. 2008;9(1):253 doi: 10.1186/1471-2105-9-253 18510760 10.1186/1471-2105-9-253 PMC2438325

[45] Nguyen-Dumont T, Pope BJ, Hammet F, Southey MC, Park DJ. A high-plex PCR approach for massively parallel sequencing. Biotechniques. 2013;55(2):69–74. doi: 10.2144/000114052 23931594 10.2144/000114052

[46] Sumabat LG, Kemerait RC, Brewer MT, editors. Host-specialized populations of *Corynespora cassiicola* causing emerging target spot epidemics in the southeastern U.S. (Abstr.). Phytopathology 107:S31 http://dxdoiorg/101094/PHYTO-107-4-S31; 2017. 30321244 10.1371/journal.pone.0205849 PMC6188889

[47] McDonald BA, Linde C. The population genetics of plant pathogens and breeding strategies for durable resistance. Euphytica. 2002;124(2):163–80.

